# Towards a unified framework for single‐cell ‐omics‐based disease prediction through AI

**DOI:** 10.1002/ctm2.70290

**Published:** 2025-04-01

**Authors:** Matteo Barberis, Jinkun Xie

**Affiliations:** ^1^ Molecular Systems Biology, School of Biosciences, Faculty of Health and Medical Sciences University of Surrey Guildford UK; ^2^ Centre for Mathematical and Computational Biology, CMCB University of Surrey Guildford UK

**Keywords:** artificial intelligence, Clinical Decision Assistant, deep learning, disease prediction, drug repurposing, machine learning, medicine, single‐cell ‐omics

## Abstract

Single‐cell omics has emerged as a powerful tool for elucidating cellular heterogeneity in health and disease. Parallel advances in artificial intelligence (AI), particularly in pattern recognition, feature extraction and predictive modelling, now offer unprecedented opportunities to translate these insights into clinical applications. Here, we propose single‐cell ‐omics‐based Disease Predictor through AI (scDisPreAI), a unified framework that leverages AI to integrate single‐cell ‐omics data, enabling robust disease and disease‐stage prediction, alongside biomarker discovery. The foundation of scDisPreAI lies in assembling a large, standardised database spanning diverse diseases and multiple disease stages. Rigorous data preprocessing, including normalisation and batch effect correction, ensures that biological rather than technical variation drives downstream models. Machine learning pipelines or deep learning architectures can then be trained in a multi‐task fashion, classifying both disease identity and disease stage. Crucially, interpretability techniques such as SHapley Additive exPlanations (SHAP) values or attention weights pinpoint the genes most influential for these predictions, highlighting biomarkers that may be shared across diseases or disease stages. By consolidating predictive modelling with interpretable biomarker identification, scDisPreAI may be deployed as a clinical decision assistant, flagging potential therapeutic targets for drug repurposing and guiding tailored treatments. In this editorial, we propose the technical and methodological roadmap for scDisPreAI and emphasises future directions, including the incorporation of multi‐omics, standardised protocols and prospective clinical validation, to fully harness the transformative potential of single‐cell AI in precision medicine.

In the last decade, the use of single‐cell ‐omics in clinical and translational medicine has attracted the attention of scientists across diverse research areas. Its ability to uncover molecular information within tissues aided the research in drug discovery,^1^ immunology^2^ and cancer research.^3^ At the same time, artificial intelligence (AI) has advanced in pattern recognition^4^ and feature extraction,^5^ and it has proved to make clinical predictions.^6^ By integrating single‐cell ‐omics through AI, we propose a unified framework for single‐cell ‐omics‐based disease prediction, i.e., the single‐cell ‐omics‐based Disease Predictor through AI (scDisPreAI). In this framework, single‐cell ‐omics data serve as input to achieve two goals: (i) generation of a comprehensive Clinical Decision Assistant (CDA) for a wide range of disease predictions along with its stage, and (ii) identification of the biomarkers associated with these predictions. Importantly, the framework has the potential to identify common biomarkers, e.g., a gene being identified as a biomarker for different diseases, thereby opening avenues for drug repurposing (Figure 1).

**FIGURE 1:**
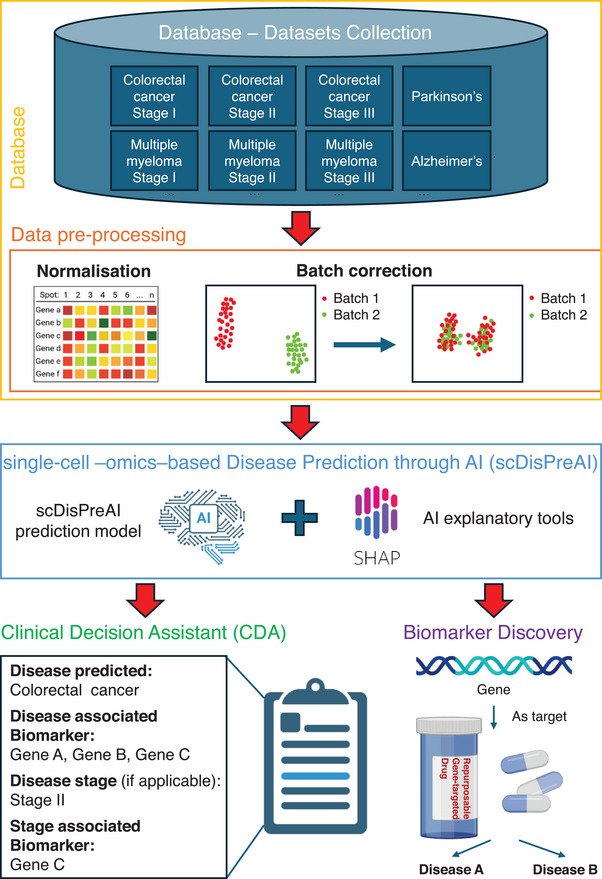
Framework of the single‐cell ‐omics‐based Disease Predictor through Artificial Intelligence (scDisPreAI). The framework consists of three main components: (1) a comprehensive database of ‐omics data for training—a large, standardised database containing diverse diseases and disease stages that serves as the foundation for training scDisPreAI. The data undergo pre‐processing, including normalisation and batch correction, ensuring standardisation across datasets before model training. (2) AI‐based Disease Predictor (scDisPreAI)—the pre‐processed data are used to train the scDisPreAI model, which predicts diseases and disease stages. AI explanatory tools (e.g., SHapley Additive exPlanations [SHAP]) identify the key genes driving the model's predictions. (3) Outcomes of scDisPreAI: (i) Clinical Decision Assistant (CDA). The model generates a report detailing the predicted disease, disease stage and the most relevant gene(s) specific for the disease stage. (ii) Biomarker discovery. The identified gene(s) serve as potential biomarkers for disease stage progression and treatment. Importantly, scDisPreAI can recognise genes consistently important across multiple diseases, enabling drug repurposing by identifying therapeutic targets shared across different disease conditions. This integrated framework enables precise disease prediction, biomarker identification and potential therapeutic insights, enhancing both clinical decision‐making and drug discovery. Created with and adapted from BioRender.com.

To train such an AI framework, we first need a large, standardised database covering a wide array of diseases and disease stages (Figure 1). This aligns with the recently envisioned clinical artificial intelligent single‐cell (caiSC) working station, designed to integrate single‐cell ‐omics and molecular data for clinical applications through AI.^7^ The dataset aims to contain single‐cell data from various diseases, and the different time points in each of the disease (e.g., disease stages). This comprehensive dataset would support the scDisPreAI workflow which is specifically designed for biomarker discovery by (i) discriminating a disease from another, and (ii) providing information about the biomarkers driving the disease progressions. Single‐cell gene expression data from diverse technologies require careful normalisation to account for variations in sequencing depth, to allow combination of different datasets. Packages such as SCANPY^8^ and Seurat^9^ can be used for normalisation. Additionally, batch effect correction is critical to remove technical variation across and within datasets introduced by different laboratories, protocols or sequencing runs, ensuring that biological rather than technical differences would drive the modelling process. Popular batch correction methods include Harmony^10^ and ComBat.^11^ While these tools are powerful, removing technical biases shall be balanced with preserving true biological signals. The gene expression data can be acquired from both scRNA‐seq and spatial transcriptomics. While scRNA‐seq typically offers single‐cell resolution, not all spatial transcriptomics platforms achieve the same level of details. From a biomarker discovery standpoint, however, the gene expression analysis does not depend on the single‐cell resolution. In the scDisPreAI framework, each tissue sample, whether sequenced by scRNA‐seq or spatial transcriptomics, is associated with a disease and a specific disease stage. Because the dataset does not specify which individual cells are diseased or at which disease stage, the same normalisation and batch correction steps shall be applied to both data types. Practically, spatial coordinates can be added to the scDisPreAI workflow, by integrating spatial pattern detection to gene expression‐based modelling. This integration enhances biomarker discovery.

Once gene expression data from different sources and disease stages has been normalised and batch‐corrected, it is ready for model training (Figure 1). As a standard practice, the dataset is typically split into training and testing subsets to allow unbiased evaluation of model performance. Given that tissues can contain thousands of genes, often measured across large numbers of cells, the data can be extremely high‐dimensional. Consequently, feature extraction or dimensionality reduction becomes essential for model training. Features may include individual genes, principal components from Principal Component Analysis (PCA),^12^ or representations derived from methods such as the t‐Distributed Stochastic Neighbor Embedding (t‐SNE)^13^ or Uniform Manifold Approximation and Projection (UMAP).^14^ The choice between deep learning and machine learning models mostly relies on the size of the dataset. If adequate data are available for each disease and disease stage, deep learning can unlock its potential by automatically learning features in a data‐driven manner. Deep learning applied to data from tissue sections typically requires a large volume of samples,^15^ often on the order of hundreds to thousands of tissue sections, to converge successfully. Conversely, when data are more limited, classical machine learning approaches can be equally effective. In these scenarios, features are typically defined based on prior biological knowledge (e.g., known biomarkers), which can yield strong performance with smaller sample sizes.

In the machine learning pipeline (Figure 2), feature extraction is often performed for each tissue sample (e.g., aggregated gene expression, principal components). Popular classifiers include Random Forest, XGBoost, CATBoost, Logistic Regression and Support Vector Machine (SVM). Tree‐based methods such as Random Forest naturally handle non‐linear relationships between input features and also perform a form of automatic feature selection.^16^ Instead, SVM methods capture linear relationships and classify input features using decision boundaries, typically hyperplanes in their default form; however, these methods can model non‐linear relationships using kernel functions.^17^ The scDisPreAI workflow can make use of a database with information about diseases and their disease stages, in which each tissue has two levels of labels: a disease label (e.g., ‘Colorectal cancer’) and a stage label (e.g., ‘Colorectal cancer Stage I’). The scDisPreAI goal is twofold: (1) to learn specific pattern of labelled data for future prediction of new tissue sections belonging to which disease (prediction task 1) and at which stage (prediction task 2), and (2) to identify biomarkers associated with disease and stage prediction. This includes discovering genes shared across different diseases, genes shared between stages of the same disease, and genes that are specific to certain diseases or stages. To achieve this, there are two possible approaches in using machine learning. The first approach involves a hierarchical setup with two levels of classifiers to achieve both prediction tasks. At the first level, a classifier is trained using the disease labels to differentiate between diseases. At the second level, a separate classifier is trained for each disease using the stage labels to predict the stages specific to that disease. This approach allows the model to focus on disease‐specific stage prediction once the disease has been identified. Alternatively, the second approach, the parallel classification, involves training two independent classifiers. One classifier uses the disease labels to predict the disease, while the other uses the stage labels across all diseases to predict the disease stage directly, irrespective of the disease itself. Both approaches have their merits, being the hierarchical approach leveraging disease‐specific information for more refined stage predictions, and the parallel approach being simpler to implement. In either case, these classifiers automatically refine features or determine decision thresholds to minimise misclassification—tree‐based models (e.g., Random Forest, XGBoost and CATBoost) set thresholds for splitting features, while linear methods (e.g., SVM and Logistic Regression) optimise a decision threshold (e.g., hyperplane) to maximise class separability.

**FIGURE 2:**
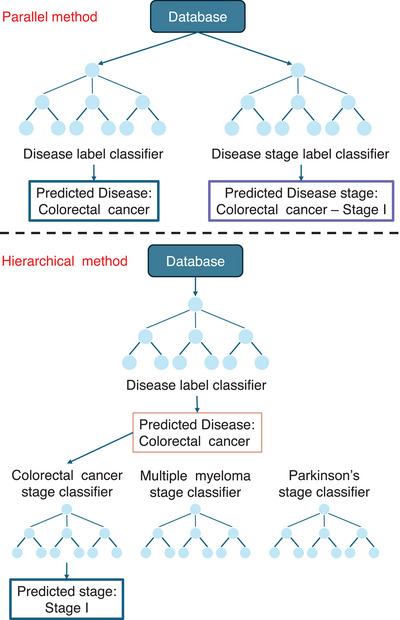
Machine learning approaches for scDisPreAI disease and disease stage prediction. The *parallel method* treats disease and disease stage as separate classification tasks, thus two independent classifiers are trained separately: one classifier predicts the disease label (e.g., ‘Colorectal cancer’), while another classifier predicts the disease stage (e.g., ‘Colorectal cancer Stage I’). Both classifiers operate in parallel and are trained to make independent predictions across all diseases and all disease stages. The *hierarchical method* models the disease‒stage relationship sequentially: a primary disease classifier first predicts the disease (e.g., ‘Colorectal cancer’); once the disease is identified, a disease‐specific stage classifier determines the corresponding stage (e.g., ‘Stage I’)—each disease has its own dedicated stage classification model, allowing for disease‐specific stage prediction. Created with and adapted from BioRender.com.

The deep learning approach bypasses the manual feature engineering by directly using the raw expression data from each tissue section as input (Figure 3). The architecture consists of three main components. The first is the input layer, which accepts the full gene expression data from a single tissue. This is followed by the feature extraction layer, where techniques such as multilayer perceptron,^18^ convolutional neural networks,^19^ graph neural networks^20^ or attention mechanisms^21^ are employed. These techniques identify and extract representative features from the data, automatically capturing the patterns needed for prediction. The output of this layer is a tissue‐level feature embedding, i.e., high‐dimensional representation of the learned features for each tissue section in the dataset. The final component is the task layer, which uses the tissue‐level feature embeddings as input to perform two tasks simultaneously. The prediction task 1 predicts the disease label, such as ‘Colorectal cancer’, while the second predicts the stage label, such as ‘Colorectal cancer Stage I’. Each task has its own loss function, and the overall loss for the model is the sum of the two loss functions. The total loss serves as the metric for model optimisation during training. Once the total loss is minimised, the model is considered trained and ready to make predictions.

**FIGURE 3:**
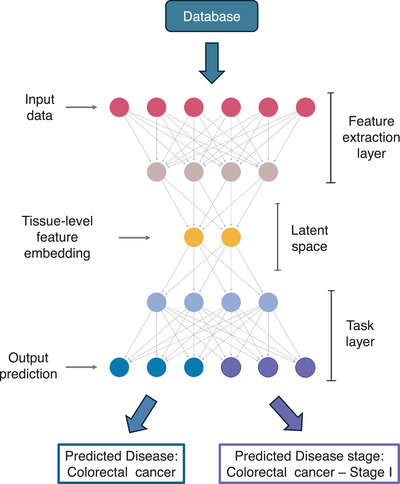
Deep learning framework for disease and disease stage prediction in scDisPreAI. Input data are sourced from the database in Figure 1, where labels correspond to whole tissue sections. The ‘feature extraction layer’ extracts tissue‐level features (e.g., gene expression patterns), generating a ‘latent space’ representation that captures meaningful embeddings of the tissue. This tissue‐level feature embedding serves as input to the ‘task layer’, which performs multi‐task learning to predict both disease and disease stage. Multi‐task learning enables the ‘task layer’ to learn features specific to each task (i.e., disease prediction and disease stage prediction) while also leveraging shared representations to improve overall prediction accuracy. Created with and adapted from BioRender.com.

Both machine learning and deep learning approaches require evaluation using metrics such as Area Under Curve (AUC) of Receiver Operating Characteristic (ROC) graph—a visual representation of model performance across all thresholds, to measure how well a parameter can distinguish between two diagnostic groups (diseased/normal)—precision, recall, accuracy and *F*1‐score to assess performance.^22^ Cross‐validation is performed during training.^23^ Metrics are evaluated across cross‐validation folds, and the mean values are used to monitor the model's performance. Once (i) the training AUC and the mean cross‐validation AUC indicate that the model is neither overfitting nor underfitting, and (ii) the metrics at individual cross‐validation folds are consistent to indicate the stability of the model, then the test subset is used to assess the model's performance on unseen data. If the model performs well on the test data, it is deemed ready for deployment and model interpretation, which indicates the important features resulting in the model's prediction. Interpretability tools, such as the widely used SHapley Additive exPlanations (SHAP),^24^ are used for both machine learning and deep learning models. SHAP is grounded in game theory and it quantifies the contribution of each gene (the feature used for model prediction) to the model's predictions for disease and disease stages (Figure 1). A positive SHAP value indicates that a gene positively contributes to the prediction, while a negative SHAP value implies a negative contribution. The magnitude of the SHAP value reflects the strength of the gene's impact on the prediction, with larger magnitudes signifying a greater influence. SHAP enables the identification of genes that are important for each prediction task, allowing these genes to serve as biomarkers. Furthermore, the tool identifies important genes (i.e., biomarkers) that are commonly shared across different diseases or stages within the same disease, thus facilitating the identification of drugs that may be repurposed to target these biomarkers. If attention mechanisms are incorporated into a deep learning model, the attention weights can similarly highlight genes that are important for each prediction task.^21^ This mechanism not only identifies task‐specific biomarkers but also has the capability to uncover common biomarkers shared across diseases or disease stages. Together, SHAP and attention mechanisms provide robust tools for understanding model predictions and extracting biologically meaningful insights.

The model that is deployed serves as a Clinical Decision Assistant (CDA) (Figure 1). Given a patient's gene expression dataset, CDA predicts the disease (if present), determines the disease stage, and highlights the associated biomarkers. The information about biomarkers can provide valuable insights to clinicians, enabling them to plan diagnostic tests and/or identifying targeted therapies that aim at improving patient outcomes. Besides its clinical use, scDisPreAI provides molecular insights into the underlying mechanisms of diseases. By leveraging a comprehensive database, such as the recently envisioned caiSC database,^7^ scDisPreAI can identify potential gene targets for repurposable drugs (Figure 1), whether for multiple diseases or different stages of a single disease condition. To generate a CDA and identify biomarkers for repurposable drugs, more studies across a diverse range of diseases and—importantly—across different time points, i.e., different disease stages, are needed to capture the dynamic molecular landscape of disease progression. However, consistency across studies remains a challenge due to experiment‐specific biases and technical variations. A standardised experimental protocol is crucial to minimising these inconsistencies, thus enhancing the robustness of cross‐experiment analysis, and ensuring data comparability. While statistical and deep learning methods have made significant progress in batch effect removal,^25^ technical variations in sample preparation, sequencing depth and library construction can still obscure biological signals. Further research is needed to refine these computational approaches while ensuring that biological signals are preserved. Ultimately, experimental standardisation is as critical as computational correction, and community‐wide adoption of standardised protocols would strengthen dataset reliability, enabling more reproducible and biologically meaningful analyses.

scDisPreAI can be extended to incorporate multi‐omics data (e.g., proteomics, metabolomics, lipidomics) in addition to transcriptomic data. In machine learning models, multiple classifiers may be trained per data type and predictions may then be combined. However, this approach may not fully capture interdependencies across different ‐omics. Instead, multimodal deep learning architectures can integrate features from each ‐omics type automatically, offering a holistic view of disease mechanisms. To further increase predictive accuracy and clinical relevance, scDisPreAI could incorporate additional data streams such as radiological imaging or patient clinical histories. Multimodal approaches that integrate these data types with single‐cell ‐omics can yield more robust predictions and guide precision medicine.

Recent efforts such as scGPT^26^ demonstrate the potential of foundation models to generate gene expression profiles, perform batch correction and predict perturbation responses. However, these efforts primarily focus on data analysis rather than direct clinical disease prediction. scDisPreAI fills this gap by offering a scalable, adaptable framework that bridges new diseases and emerging data modalities, integrating the worlds of single‐cell ‐omics, large‐scale AI models, and clinical translation. In summary, scDisPreAI provides a unified, powerful framework for disease and disease stage prediction rooted in single‐cell ‐omics. By integrating robust AI models, whether machine learning or deep learning, and leveraging interpretability tools such as SHAP and attention mechanisms, scDisPreAI is able to (i) offer precise clinical disease classifications, and (ii) elucidate key disease biomarkers by identifying genes target for repurposable drugs that may have broad therapeutic implications. Moving forward, expanding the disease database, standardising experimental protocols, incorporating multi‐omics data and validating the workflow in prospective clinical studies will be crucial steps towards realising the full promise of single‐cell AI in clinical and translational medicine. Embracing this integrated paradigm holds the potential to revolutionise clinical diagnostics, accelerate drug discovery and ultimately improve patient care on a global scale.

## AUTHOR CONTRIBUTIONS

Matteo Barberis conceived and formulated the idea and framework, drawn the logic of the manuscript and wrote the manuscript. Jinkun Xie contributed to the idea and logic of the manuscript, helped with the drafting of figures and wrote the manuscript.

## CONFLICT OF INTEREST STATEMENT

The authors declare they have no conflicts of interest.

## ETHICS STATEMENT

Not applicable.

## DECLARATION OF GENERATIVE AI AND AI‐ASSISTED TECHNOLOGIES

ChatGPT was used to improve the readability and language in some parts of the text. After using this tool, the authors reviewed and edited the content as needed and take full responsibility for the content of the publication.

## References

[1] Van De Sande B , Lee JS , Mutasa‐Gottgens E , et al. Applications of single‐cell RNA sequencing in drug discovery and development. Nat Rev Drug Discov. 2023;22(6):496‐520. doi:10.1038/s41573-023-00688-4 37117846 PMC10141847

[2] Papalexi E , Satija R . Single‐cell RNA sequencing to explore immune cell heterogeneity. Nat Rev Immunol. 2018;18(1):35‐45. doi:10.1038/nri.2017.76 28787399

[3] Baysoy A , Bai Z , Satija R , Fan R . The technological landscape and applications of single‐cell multi‐omics. Nat Rev Mol Cell Biol. 2023;24(10):695‐713. doi:10.1038/s41580-023-00615-w 37280296 PMC10242609

[4] Abiodun OI , Jantan A , Omolara AE , et al. Comprehensive review of artificial neural network applications to pattern recognition. IEEE Access. 2019;7:158820‐158846. doi:10.1109/ACCESS.2019.2945545

[5] Elharrouss O , Akbari Y , Almadeed N , et al. Backbones‐review: feature extractor networks for deep learning and deep reinforcement learning approaches in computer vision. Comput Sci Rev. 2024;53:100645. doi:10.1016/j.cosrev.2024.100645

[6] Khalifa M , Albadawy M . Artificial intelligence for clinical prediction: exploring key domains and essential functions. Comput Methods Programs Biomed Update. 2024;5:100148. doi:10.1016/j.cmpbup.2024.100148

[7] Wang X , Powell CA , Ma Q , et al. Clinical and translational mode of single‐cell measurements: an artificial intelligent single‐cell. Clin Transl Med. 2024;14(9):e1818. doi:10.1002/ctm2.1818 39308059 PMC11417141

[8] Wolf FA , Angerer P , Theis FJ . SCANPY: large‐scale single‐cell gene expression data analysis. Genome Biol. 2018;19(1):15. doi:10.1186/s13059-017-1382-0 29409532 PMC5802054

[9] Satija R , Farrell JA , Gennert D , et al. Spatial reconstruction of single‐cell gene expression data. Nat Biotechnol. 2015;33(5):495‐502. doi:10.1038/nbt.3192 25867923 PMC4430369

[10] Korsunsky I , Millard N , Fan J , et al. Fast, sensitive and accurate integration of single‐cell data with harmony. Nat Methods. 2019;16(12):1289‐1296. doi:10.1038/s41592-019-0619-0 31740819 PMC6884693

[11] Leek JT , Johnson WE , Parker HS , Jaffe AE , Storey JD . The sva package for removing batch effects and other unwanted variation in high‐throughput experiments. Bioinformatics. 2012;28(6):882‐883. doi:10.1093/bioinformatics/bts034 22257669 PMC3307112

[12] Pearson K . LIII. On lines and planes of closest fit to systems of points in space. Lond Edinburgh Dublin Philos Mag J Sci. 1901;2(11):559‐572. doi:10.1080/14786440109462720

[13] van der Maaten L , Hinton G . Visualizing data using t‐SNE. J Mach Learn Res. 2008;9(86):2579‐2605.

[14] McInnes L , Healy J , Melville J . UMAP: uniform manifold approximation and projection for dimension reduction. arXiv. 1802.03426v3. doi:10.48550/arXiv.1802.03426

[15] Khened M , Kori A , Rajkumar H , et al. A generalized deep learning framework for whole‐slide image segmentation and analysis. Sci Rep. 2021;11(1):11579. doi:10.1038/s41598-021-90444-8 34078928 PMC8172839

[16] Rigatti SJ . Random Forest. J Insur Med. 2017;47(1):31‐39. doi:10.17849/insm-47-01-31-39.1 28836909

[17] Hearst MA , Dumais ST , Osuna E , et al. Support vector machines. IEEE Intell Syst. 1998;13(4):18‐28. doi:10.1109/5254.708428

[18] Bourlard HA , Morgan N . Feature extraction by MLP. In: Bourlard HA , Morgan N , eds. Connectionist Speech Recognition: A Hybrid Approach. USA: Springer; 1994:253‐263. doi:10.1007/978-1-4615-3210-1_14

[19] Jogin M , Mohana X , Madhulika MS , et al. Feature extraction using convolution neural networks (CNN) and deep learning. In: 2018 3rd IEEE International Conference on Recent Trends in Electronics, Information & Communication Technology (RTEICT) . 2018:2319‐2323. doi:10.1109/RTEICT42901.2018.9012507

[20] Acharya DB , Zhang H . Feature selection and extraction for graph neural networks. In: Proceedings of the 2020 ACM Southeast Conference (ACMSE’20) . 2020:252‐255. doi:10.1145/3374135.3385309

[21] Wang Y , Wang Y . Feature extraction by using attention mechanism in text classification. In: Qin P , Wang H , Sun G , Lu Z , eds. Data Science. Springer; 2020:77‐89. doi:10.1007/978-981-15-7984-4_6

[22] Novaković JD , Veljović A , Ilić SS , et al. Evaluation of classification models in machine learning. Theory Appl Math Comput Sci. 2017;7(1):39‐46.

[23] Raschka S . Model evaluation, model selection, and algorithm selection in machine learning. arXiv. 1811.12808v1. doi:10.48550/arXiv.1811.12808

[24] Lundberg SM , Lee SI . A unified approach to interpreting model predictions. *arXiv*. 1705.07874v2. doi:10.48550/arXiv.1705.07874

[25] Tran HTN , Ang KS , Chevrier M , et al. A benchmark of batch‐effect correction methods for single‐cell RNA sequencing data. Genome Biol. 2020;21(1):12. doi:10.1186/s13059-019-1850-9 31948481 PMC6964114

[26] Cui H , Wang C , Maan H , et al. scGPT: toward building a foundation model for single‐cell multi‐omics using generative AI. Nat Methods. 2024;21(8):1470‐1480. doi:10.1038/s41592-024-02201-0 38409223

